# Dual Left Anterior Descending Coronary Artery

**DOI:** 10.4103/1995-705X.73216

**Published:** 2010

**Authors:** Muhammad Dilawar

**Affiliations:** * Pediatric Cardiology Section, Department of Cardiology and Cardiothoracic Surgery, Hamad Medical Corporation, Doha, Qatar

## CONGENITAL CORONARY ARTERY ABNORMALITIES

In this issue, Bhatia *et al*, report a rare congenital anomaly — Type IV Dual left anterior descending (LAD) artery. As mentioned in this case report, coronary artery anomalies are rare in normal hearts and slightly more common in congenital heart diseases.

The most common anomaly (accounting for about one-third of all major coronary arterial anomalies) is the origin of the left circumflex coronary artery from the right main coronary artery. It crosses behind the aorta to reach its normal territory of supply, and has no clinical significance. This artery may be compressed if both mitral and aortic prosthetic fixation rings are implanted, and such arteries may have an unusually high incidence of coronary atheroma.

Much less common (accounting for 1 – 2% of the major coronary arterial anomalies) but of great clinical significance, is the origin of the left main coronary artery from the right sinus of the valsalva. It may cross behind the aorta, in between the aorta and the right ventricular outflow tract (RVOT) or anterior to the RVOT. The course that passes between the two great vessels (aorta and RVOT) has been associated with sudden death in all age groups. Several of these patients have syncope or chest pain with exercise and then suddenly die with vigorous exercise.

In some patients, the left anterior descending coronary artery originates from the right sinus of the valsalva or from the right main coronary artery. This anomaly is rare in the absence of congenital heart disease, but is seen in 4 – 5% of the Tetralogy of Fallot patients. The artery usually passes in front of the RVOT and is surgically important in cases of ventriculotomies. To save the artery, the surgeon has to make a ventriculotomy incision either parallel to, above or below the artery, and if not possible, then right ventricle to pulmonary artery conduit placement is needed.

The origin of the right main coronary artery from the left sinus of the valsalva is relatively common, accounting for almost 30% of all major coronary artery anomalies. It courses between the aorta and the RVOT to reach the right side of the atrioventricular groove. It is generally benign, but there are case reports of myocardial ischemia, infarction, and sudden death.

In up to 20% of all major coronary artery anomalies, a single coronary artery arises either from the right or left sinus of the valsalva and then branches into left and right coronary arteries. Most single coronary arteries produce no symptoms in the absence of severe atheroma (which is clearly more serious, when there is only one main coronary artery supplying the whole heart), but a small number of premature deaths have been reported in patients in whom a major artery has crossed between the aorta and the RVOT.

Although congenital coronary artery anomalies are rare in normal hearts, they can still be a potential cause of exercise-induced syncope, chest pain, and sudden death. Therefore, if a young patient has symptoms of exercise-induced syncope or chest pain, a computed tomography (CT) angiogram or coronary arteriography may be helpful. Congenital heart patients like Tetralogy of Fallot need careful preoperative evaluation to rule out any abnormal coronary artery coursing anterior to RVOT so that surgeon can plan RVOT resection/conduit placement accordingly.

